# Applicability and outcome of laparoscopic adrenalectomy for large tumours

**DOI:** 10.11604/pamj.2018.31.23.15153

**Published:** 2018-09-11

**Authors:** Alila Mohammed, Hamdane Amine, Sara El Atiq, Bounoual Mohammed, Mouaqit Ouadii, Mazaz Khalid, Ait Taleb Khalid, Ousadden Abdelmalek

**Affiliations:** 1 Department of Visceral and Endocrine Surgery, Hassan II University Hospital, Fez, Morocco; 2 Department of Urology, Hassan II University Hospital, Fez, Morocco; 3 Faculty of Medicine and Pharmacy, Sidi Mohamed Benabdellah University, Fez, Morocco

**Keywords:** Adrenalectomy, laparoscopy, large tumour

## Abstract

Laparoscopic adrenalectomy has been shown to be as safe and effective as conventional open surgery for small and benign adrenal lesions. With increasing experience with laparoscopic adrenalectomy, this approach has become the procedure of choice for the majority of patients requiring adrenalectomy. In our department, from 2011 to 2016, a total of 28 patients with 31 adrenal tumours underwent laparoscopic adrenalectomy regardless of tumour size. Our policy in the department is to exclude adrenal tumours that are potentially malignant or metastatic adrenal tumours for laparoscopic resection. In this a retrospective study, we divided patients into two groups according to tumour size: < 5 or ≥ 5 cm, which was considered as the definition of large adrenal tumours. We compared demographic data and per- and postoperative outcomes. There was no statistical difference between the two groups for per-operative complications (16,6% vs 18,75% , P = 0.71), postoperative complications (16,6% vs 18,75% , P = 0.71), postoperative length of hospital stay (5 vs 8 days P = 0.40), mortality (0% vs 0%) or oncologic outcomes: recurrence and metastasis (8.3% vs 6.25% P = 0.70). The only statistical difference was the operating time, at a mean (SD) 194 (60) vs 237 (71) min (P = 0.039) and the conversion rate (0% vs 12.5% P < 0.01). Laparoscopic adrenalectomy can be done for all patients with adrenal tumours regardless of tumour size, even it needs more time for large tumour but appears to be safe and feasible when performed by experienced surgeons.

## Introduction

Laparoscopic adrenalectomy has become the preferred technique due to quick recovery, short hospital stay, less pain and better cosmetics since its introduction in 1992 [1]. The main debate in the literature involves the surgical management of patients with large adrenal tumours. Although many studies have shown that large tumours are no longer a contraindication for laparoscopic adrenalectomy [2, 3] some authors reported laparoscopic approach for large tumours is not feasible due to the increased risk of malignancy, especially for the tumours that show infiltration to surrounding structures on computerized tomography (CT), which can also bring other risks as peritoneal dissemination or port site recurrence [4]. Based on our experience, we have been favouring laparoscopic approach in patients with adrenal tumours regardless of tumour size. The aim of this study was to evaluate the safety and efficacy of laparoscopy for large adrenal tumours by comparing the outcomes of laparoscopic adrenalectomy for tumours larger than 5 cm with those smaller than 5 cm.

## Methods

### Patients

The study included patients who underwent laparoscopic adrenalectomy between January 2011 to December 2016 at the Department of Visceral and Endocrine Surgery, Hassan II University Hospital, Fez, Morocco. The patients were divided into two study groups according to tumour size using preoperative imaging. Group 1 included patients with adrenal tumours smaller than 5cm and group 2 included larger than 5cm, which was considered as the definition of large adrenal tumours. We reviewed data on age, gender, American Society of Anaesthesiology (ASA) score, preoperative diagnosis, tumour size, operating time, conversion to open surgery, morbidity and mortality. We used the Clavien-Dindo score to classify postoperative complications. We considered a *p* < 0.05 to indicate statistical significance. Those patients with pre-operative imaging features of advanced malignancy, such as tumour invasion of the surrounding structures, systemic metastases or the requirement of additional open surgery were routinely performed open adrenalectomy and excluded from the study.

### Surgical technique

Our techniques for laparoscopic adrenalectomy have been described in detail before [5]. All the procedures were performed by the 4 senior authors (Ousadden Abdelmalek, Mouaqit Ouadii, Mazaz Khalid and Ait taleb Khalid).

### Technical aspects of laparoscopic adrenalectomy

We prefer the transperitoneal lateral decubitus approach, as the best for maximal exposure of the gland and adjacent organs and vessels. With four ports for a unilateral lesion and seven ports for bilateral adrenal tumours with the same epigastric port. Pneumoperitoneum is maintained at 12 mmHg. For dissection we use a monopolar or bipolar scalpel, occasionally ultracision or Ligasure when available.

### Right adrenalectomy

The right triangular ligament and the retroperitoneal liver attachments are cauterized and divided to allow liver retraction and expose the upper limits of the tumor. After dividing the retroperitoneum, the inferior vena cava (IVC) is identified and dissected from the tumor. The periadrenal fat is gently pushed upwards. The adrenal vein is subsequently identified, dissected, double-clipped and divided. The adrenal gland is then dissected from the rest of the adjacent structures, artery and an eventual accessory adrenal vein is ligated as we advance in dissection.

### Left adrenalectomy

The left colonic flexure is always mobilized in large tumors and the left upper renal pole exposed. The splenic attachments are cauterized and divided and the tail of the pancreas identified. The spleen is further mobilized until the stomach is visualized. Gerota´s fascia is then opened, the adrenal gland identified, and the adrenal vein dissected, double clipped and divided. The renal vein is occasionally identified prior to adrenal vein clipping. The rest of adrenal tumour is dissected from the surrounding structures and other additional adrenal branches are coagulated or clipped from inferior phrenic vessels. The specimen is extracted by a sub costal incision or incision joining two ports, in a retrieval bag. A drain is placed using the lateral port.

## Results

**Figure 1:**
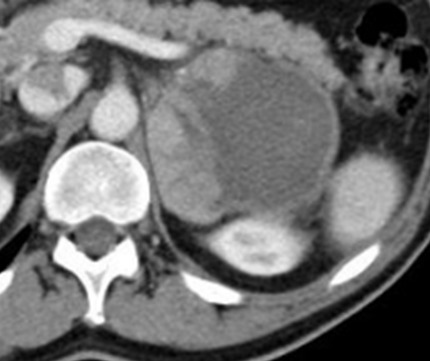
Abdominal CT scan showing left adrenal tumour of 9 cm

**Figure 2:**
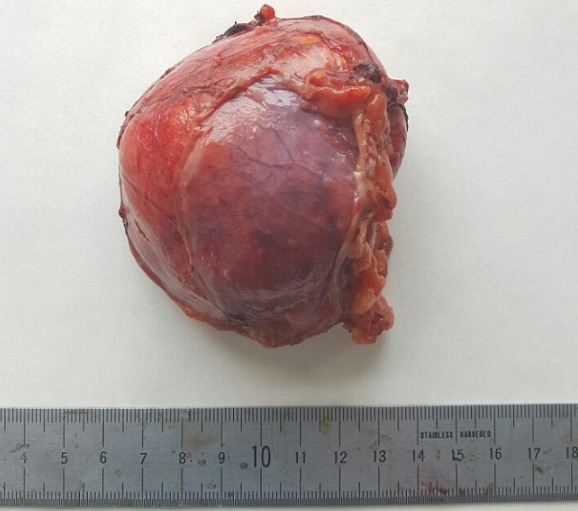
Specimen’s picture of adrenal tumour of 8x12 cm

**Figure 3:**
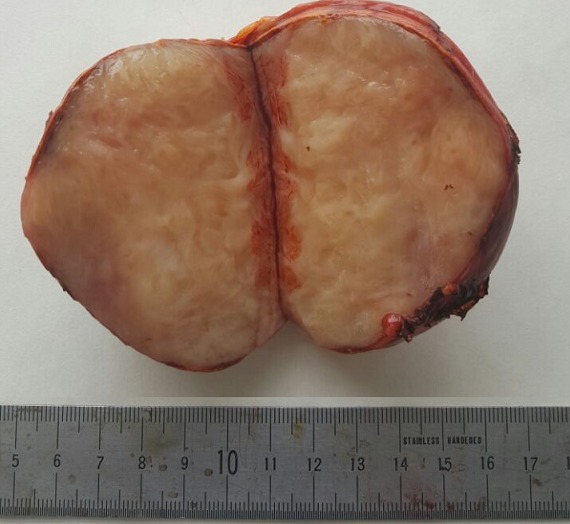
Picture of the specimen divided in two

From January 2011 to December 2016, a total of 28 patients with 31 adrenal tumours underwent laparoscopic adrenalectomy at the Department of Visceral and Endocrine Surgery, Hassan II University Hospital, Fez, Morocco. Patient demographics are displayed in Table 1. The mean age of all patients was 37 years (range 21 to 71). The male to female ratio was approximately 0,27:1. Eleven LAs were performed on the left, 14 on the right, and 3 patients underwent bilateral adrenalectomies (Figure 1, Figure 2 and Figure 3). The study groups were similar for age, gender and tumour side Table 1. The mean ages were 39 ± 12 years in group 1 and 31 ± 11 in group 2. Mean tumour size of group 1 was 4.2 cm (2-5) and 8.3 cm (5-14) in group 2. Except for the indication and ASA score. There were more phaeochromocytoma in the large-tumour group (73%, *P* = 0.03) (Table 1).

**Table 1:** Demographic data of the two groups

Variables	Small tumour < 5cm	Large tumour ≥ 5cm	p	X_2_
Number of patients	12	16	0,4502	0,57
Mean (SD) age years	39 (12)	31 (11)	0,3401	0,91
Male, n (%)	3 (25)	3 (18,75)	0,3449	0,89
ASA score, n (%)				
1	7 (58,3)	12 (75)		
2	4 (33,3)	2 (12,5)		
3	1 (8,3)	2(12,5)		
Mediane tumour size cm	4,2	8,3	0,247	1,34
**Localisation n (%)**				
Left	5(42)	6 (37,5)	0,613	0,2547
Right	5 (42)	9 (56,25)	0,150	2,066
Bilateral	2 (16)	1(6,25)	0,0303	4,688
**Indication n (%)**				
pheochromocytoma	7 (58,3)	6 (37,5)	0,0336	4,5
Adenoma cushing’s	3 (25)	2 (12,5)	0,0412	4,16
Aldosteronoma	1(8,3)	2 (12,5)	0,3571	0,841
Incidentaloma	1 (8,3)	6 (12,5)	<0,001	18,61

Comparing the two groups of patients, we found that the median operative time was longer in the large-tumour group, at a mean (SD) of 237 (71) vs 194 (60) min (*P* = 0.0399) with a difference statistically significant for the conversion rate (0% vs 12.5%, *P* < 0.001), in fact the open conversion occurred for difficulties regarding dissection. But, there were no statistical differences between the groups for per-operative complications (16,6% vs 18,75%, P = 0.71) (Table 2) or postoperative complications (16,6% vs 18,75% , *P* = 0.71). There were two (16.6%) complications in group 1 including wound infections and 3 (18.75%) in group 2 including 2 wound infections and 1 intra abdominal hematoma (Table 3). But there was no mortality. The mean hospital stay was similar between study groups with (5 vs 8 days, P = 0.40). The final pathologic examination of the specimens revealed only one adrenocortical carcinomas in group 1, versus one in group 2 (8.3% vs 6.25% days P = 0 .70) (Table 4). The mean follow-up period of the patients were 28 ± 10 months with no significant difference between study group 1 and 2 according to oncologic outcomes: recurrence and metastasis (8.3% vs 6.25% P = 0.70) (Table 4).

**Table 2:** Comparison of per operative complications between the two groups

Variables	Small tumour < 5cm n=12	Large tumour ≥ 5cm n=16	P	X_2_
Per operative complications	2 (16,6)	3 (18,75)	0,71	0,1307
Per operative hypertension	2 (16,6)	1 (6,25)	0,03	4,688
Hypoglycemia	0	1 (6,25)	0,0124	6,25
Minor liver or spleen tears	0	0	-	-
Bleeding	0	1(6,25)	0,0124	6,25
cardiovascular events (myocardial	0	0	-	-
infarction or cerebrovascular accident				

**Table 3:** Results of statistical comparison of operative time and post-operative outcomes between the two groups

Variables	Small tumour < 5cm	large tumour > 5cm	P	X_2_
Number of patients	12	16	0,4502	0,57
Mean operative time, min (SD)	194 [90-280]	227 [173-310]	0,0399	4,22
Conversion, n (%)	0 (0)	2 (12,5)	< 0,001	12,5
Post-operative complications, n (%)	2 (16,6)	3 (18,75)	0,71	0,1307
Intra-abdominal hematoma Wound Infection Deep vein thrombosis	0 2 (16,6) 0	1 (6,25) 2 (12,5) 0	0,012 0,44 -	6,15 0,577 -
Median (range) post-operative stay, days	5 (3-8)	8 (3-15)	0,40	0,69
Hospital Mortality, n (%)	0	0	-	-

**Table 4:** Results of statistical comparison of oncologic outcomes between the two groups

Variables	Small tumour < 5cm n=12	Large tumour ≥ 5cm n=16	P	X_2_
Malignant tumour	1 (8,3)	1 (6,25)	0,70	0,144
Peritoneal dissemination	0	0	-	-
Recurrence	0	0	-	-
Metastasis	1 (8,3)	1(6,25)	0,70	0,144

## Discussion

Laparoscopic adrenalectomy has become the gold standard in management of most adrenal masses [1, 2]. In fact, over the last 2 decades, retrospective comparison studies have illustrated the superiority of the laparoscopic approach over the conventional open procedure for the removal of benign functioning and nonfunctioning tumors of the adrenal gland. Laparoscopic procedures are associated with decreased hospitalization time; less operative blood loss; less postoperative discomfort, pain and need for analgesics; faster postoperative recovery; earlier return to everyday activities and diet; and lower overall costs [5]. Based on these considerations, the indications for this technique have been vastly expanded and laparoscopic adrenalectomy may even be performed, in select cases, on an outpatient basis [5].

Three issues are of utmost importance while dealing with large adrenal tumour. First is the intra-operative technical difficulty due to distorted anatomy and overhanging on surrounding important vascular pedicles, the second being the risk of dealing with a malignant neoplasm. The third issue is retrieval of these large tumours without intra peritoneal spillage. In our present study, we considered tumours of ≥ 5cm as large. Defining ‘large’ adrenal tumours is subject to controversy. Some recent authors suggest 6 or 8 cm as thresholds [5, 6], but most authors support the size of 5 cm as large because of the risk of malignancy in larger tumours [7, 8].

In the literature, the dissection time and the conversion rate were dependent on the characteristics and size of the tumour, first by disturbing the surrounding anatomy of the adrenal gland and secondly because the surface of dissection is also increased. The mean operative time can be improved with experience (learning curve) [6, 9]. Contrary to operative time and conversion to open surgery, our data revealed no significant difference in immediate outcomes of LA for patients with large adrenal tumours compared to smaller tumours and there was no association between tumour size and hospital stay, oncologic or late outcomes of LA. The morbidity rate ranges from 6% to 16% and these are mostly minor complications [7, 8]. In our present study, no major complications occurred in patients with large tumours, probably because they were operated towards the latter period of our experience and we think that the surgeons were more careful regarding the size of the lesion. There was no difference in postoperative hospital stay between the two groups. Patients with large adrenal tumours can benefit from a short postoperative hospital stay [4, 8, 10].

In our data, oncologic outcomes were reported to be similar between study groups. But in the littereatue [11], there are still concerns regarding the ability of the laparoscopic approach to totally remove primary malignant lesions that are supported by some cases of local recurrence and peritoneal tumor dissemination following laparoscopic approaches for primary malignancies. Recurrence may be due to incomplete resection or capsular disruption of the tumor during manipulation of the adrenal mass [12, 13].

In our daily practice, we believe that any sign of suspected malignancy on pre-operative imaging should result in open surgery, so the interpretation of radiologic characteristics is a cornerstone of preoperative assessment of large masses, because open surgery remains the preferred procedure when malignancy is suspected. Tumor size is a good index but cannot be used as an absolute predictor of malignancy [9]. It has been estimated that the risk for cancer in adrenal tumors > 6cm is 1 in every 60 adrenalectomies performed ie 1.67% [10]. On the other hand, 13.5% of adrenocortical carcinomas were diagnosed in patients with adrenal tumors < 5cm [4]. Moreover, computed tomography may be associated with approximately a 40% underestimation of adrenal tumor size compared with the actual size determined in the histological examination [8]. Despite the improvement in imaging techniques, they lack enough accuracy to exclude primary malignancy. An initial laparoscopic approach can be used to establish a diagnosis and conversion to the open technique is mandatory if curative resection cannot be performed. The sole widely accepted absolute contraindication for minimally invasive techniques in adrenal lesions is the presence of large primary carcinomas with or without local invasion of nearby structures and/or metastasis to periaortic lymph nodes [12]. Large but well-encapsulated metastatic adrenal masses without evidence of local invasion can be removed laparoscopically [13].

## Conclusion

Without pre-operative suspicion of malignancy, laparoscopic technique is safe and feasible for adrenal tumours regardless of tumour size and can be performed by general surgeons with laparoscopic experience even in developing countries.

### What is known about this topic

- Laparoscopic adrenalectomy has been shown to be as safe and effective as conventional open surgery for small and benign adrenal lesions.

### What this study adds

- Laparoscopic technique is safe and feasible for adrenal tumours regardless of tumour size, if the tumour isn’t locally invasive on pre-operative imaging.

## Competing interests

The authors declare no competing interests.

## Authors’ contributions

Alila Mohammed wrote the paper and gathered referenced data. Hamdane Amine, El Atiq Sara, Bounoual Mohammed: gathered referenced data, contributed equally in organizing them, and reviewed the draft. Mouaqit Ouadii, Mazaz Khalid, Ait Taleb Khalid: participated in the follow up. Ousadden Abdelmalek: reviewed the final paper. All authors have read and agreed to the final manuscript.

## References

[1] Gagner M, Lacroix A, Bolté E (1992). Laparoscopic adrenalectomy in Cushing's syndrome and pheochromocytoma. N Engl J Med.

[2] Carter YM, Mazeh H, Sippel RS, Chen H (2012). Safety and feasibility of laparoscopic resection for large (≥ 6CM) pheochromocytomas without suspected malignancy. Endocr Pract. September.

[3] Zografos GN, Farfaras A, Vasiliadis G, Pappa T, Aggeli C, Vassilatou E (2010). Laparoscopic resection of large adrenal tumors. JSLS.

[4] Kazaryan AM, Mala T, Edwin B (2001). Does tumor size influence the outcome of laparoscopic adrenalectomy?. J Laparoendosc Adv Surg Tech A.

[5] Erbil Y, Barbaros U, Karaman G, Bozbora A, Ozarmagan S (2009). The change in the principle of performing laparoscopic adrenalectomy from small to large masses. Int J Surg.

[6] Henry JF, Sebag F, Iacobone M, Mirallie E (2002). Results of laparoscopic adrenalectomy for large and potentially malignant tumors. World J Surg.

[7] Al-Zahrani HM (2012). Laparoscopic adrenalectomy: an update. Arab J Urol.

[8] Hemal AK, Singh A, Gupta NP (2008). Whether adrenal mass more than 5 cm can pose problem in laparoscopic adrenalectomy - An evaluation of 22 patients?. World J Urol.

[9] Ramacciato G, Mercantini P, La Torre M, Di Benedetto F, Ercolani G, Ravaioli M (2008). Is laparoscopic adrenalectomy safe and effective for adrenal masses larger than 7 cm?. J Surg Endosc.

[10] Parnaby CN, Chong PS, Chisholm L, Farrow J, Connell JM, O’Dwyer PJ (2008). The role of laparoscopic adrenalectomy for adrenal tumours of 6 cm or greater. Surg Endosc.

[11] Rosoff JS, Raman JD, Del Pizzo JJ (2008). Laparoscopic adrenalectomy for large adrenal masses. Curr Urol Rep.

[12] Miller BS, Ammori JB, Gauger PG, Broome JT, Hammer GD, Doherty GM (2010). Laparoscopic resection is inappropriate in patients with known or suspected adrenocortical carcinoma. World J Surg.

[13] Brix D, Allolio B, Fenske W, Agha A, Dralle H, Jurowich C (2010). Laparoscopic versus open adrenalectomy for adrenocortical carcinoma: Surgical and oncologic outcome in 152 patients. Eur Urol.

